# Extensive eye-oral-bronchial mucosal nodules with eosinopgillia: a rare case report and literature review

**DOI:** 10.1186/s12890-020-01340-2

**Published:** 2020-11-12

**Authors:** Lujin Wu, Qianru Leng, Yan Wang, Daowen Wang, Danlei Yang

**Affiliations:** 1 Division of Cardiology, Department of Internal Medicine, Tongji Hospital, Tongji Medical College, Huazhong University of Science and Technology, Wuhan, China; 2 Hubei Key Laboratory of Genetics and Molecular Mechanism of Cardiologic Disorders, Wuhan, China; 3 Department of Respiratory and Critical Care Medicine, Tongji Hospital, Tongji Medical College, Huazhong University of Science and Technology, Wuhan, 430030 People’s Republic of China

**Keywords:** Mucosal nodules, Eosinophilia, Glucocorticoid, Case report

## Abstract

**Background:**

Mucosal nodules can be caused by infection, inflammation and neoplastic disease. Many noninfectious diseases, such as eosinophilia, amyloidosis, sarcoidosis, Wegener’s granuloma, langerhans cell histiocytosis etc., are associated with the formation of multisytem mucosal nodules, especially significant bronchial lesions. Detailed medical history, comprehensive metabolic profile, biopsy specimen and imaging examinations are required for differentiating among these disorders. The process of diagnosis and treatment of our patient’s mucosal nodules was challenging, which could be helpful to similar cases.

**Case presentation:**

We represent a case of a 29-year-old woman with plentiful nodules of unknown origin on extensive mucous membranes. Biopsy specimen reports inflammatory lesions with large numbers of neutrophils, lymphocytes, and varying degrees of eosinophils. Treatment of anti-infection, anti-tussive and anti-allergic was ineffective, but glucocorticoid showed great improvement to her symptoms.

**Conclusion:**

We experienced a rare case with plentiful nodules of unknown origin on extensive mucous membranes. She may be a specific phenotype of eosinophilia or may be a novel multisystem disease with respiratory system as the primary symptom. The diagnosis of our patient remains unclear, but tentative glucocorticoid therapy was beneficial.

## Background

Rare diseases are conditions that affect a small proportion of the population; the definition of rare disease varies from country to country due to the influence of region and population base. In the USA they are described as affecting fewer than 200,000 people (https://rarediseases.info.nih.gov), and in Europe as affecting fewer than one in 2000 (https://www.eurordis.org/about-rare-diseases). In China, the incidence rate is defined as less than one in 10,000 newborns and less than one in 500,000 people [1]. There are many kinds of rare diseases of respiratory system, which are easy to be missed and misdiagnosed in clinic. Current reports include: langerhans cell histiocytosis, eosinophilia, pulmonary alveolar proteinosis (PAP), lymphangioleyomiomatosis (LAM), bronchial amyloidosis, Wegener’s granuloma, Kartagener syndrome, bronchial mucosal tuberculosis, osteomalacia, etc. [2–4]. We treated a patient with recurrent cough and expectoration, accompanying with multiple nodules in the oral mucosa and ophthalmic conjunctiva. Bronchoscopy, laryngoscopy and gastroduodenoscopy confirmed that widespread and dense nodules were distributed in the trachea, left and right bronchus, posterior pharyngeal wall and esophagus. In addition, the patient had congenital cleft palate and dextroversion of heart. Pathological and etiological examination were mainly characterized by inflammatory lesions with large numbers of neutrophils, lymphocytes, and varying degrees of eosinophils. The examination results also excluded specific pathogen infection, granulomatous disease, amyloidosis, calculosis, and neoplastic diseases. Treatment with a variety of antibiotics was invalid but tentative glucocorticoid therapy was beneficial. This patient may be a specific phenotype of eosinophilia or may be a novel multisystem disease with respiratory system as the primary symptom.

## Case presentation

**Fig. 1:**
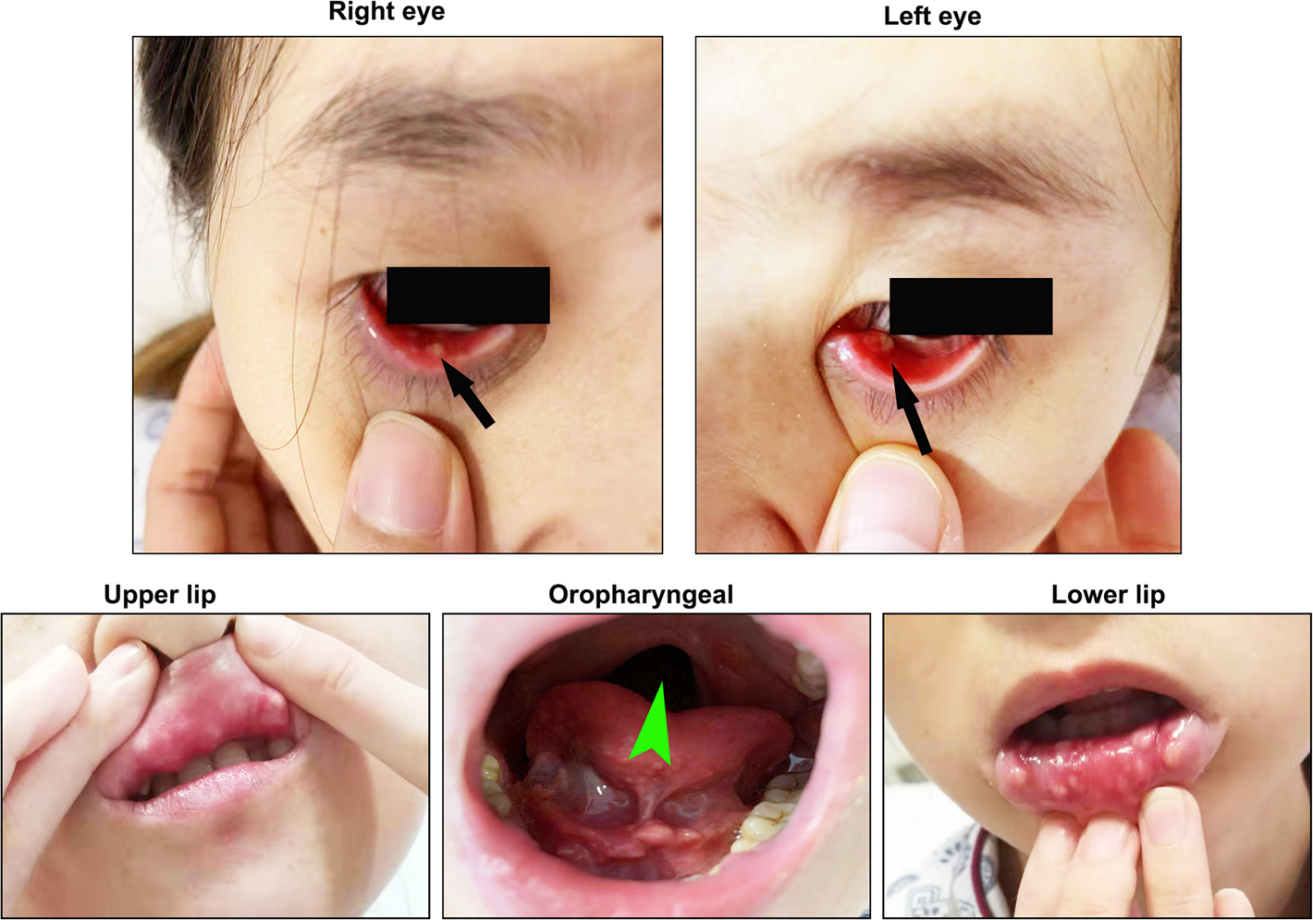
Plentiful nodules on the eyelids and oral mucosa of our patient. She also had palatoschisis in the upper jaw

A 29-year-old non-smoking woman was referred to our department for repeated coughing and expectoration for more than 4 months and gradual appearance of eye and mouth nodules. At start in May 2019, the patient felt pain in her eyes with pustules developing around them. Later the patient began to experience pharyngeal itching, cough, expectoration, fatigue and occasional chest distress. There was no history of fever, night sweats, loss of appetite or weight, joint pains, rash, hair fall, oral ulcer, photophobia and decreased vision. She received intravenous antibiotics at local community hospital for a week but the curative effect was poor. On July 22, 2019, she was firstly admitted to Wuhan Union Hospital on suspicion of bronchial asthma. Physical examination revealed that both lungs had sporadic wheezing. Her ESR was 103 mm/H (normal range 0–20 mm/H) and IgE was 159.30 IU/ml (normal range 0–100 IU/ml). Pulmonary function examination showed severe mixed pulmonary ventilation dysfunction and bronchial dilation test (negative). Chest CT showed that the right upper lobe had minute nodules; the right lower lobe had proximal segmental lung insufficiency; the right paraspinal pleura was slightly thickened; the mediastinal lymph nodes were increased; and the wall of the esophagus was limited and slightly thickened. The patient was treated with piperacillin sulbactam sodium, loratadine and montelukast sodium. After a few days of symptom remission, her cough and expectoration aggravated again. On presentation to our institution on September 12, 2019, she had plentiful nodules on her eyelids and oral mucosa with uranoschisis in the upper jaw (Fig. 1).

Past medical history: in 2012, she underwent reduction surgery in Hubei provincial people’s hospital due to congenital dextrocardia. She denied any medical histories about tuberculosis, hepatitis, hypertension, diabetes and rheumatic immune diseases. The patient also had no history of allergies or blood transfusions. The patient declared no family history of any relevant conditions.

**Fig. 2:**
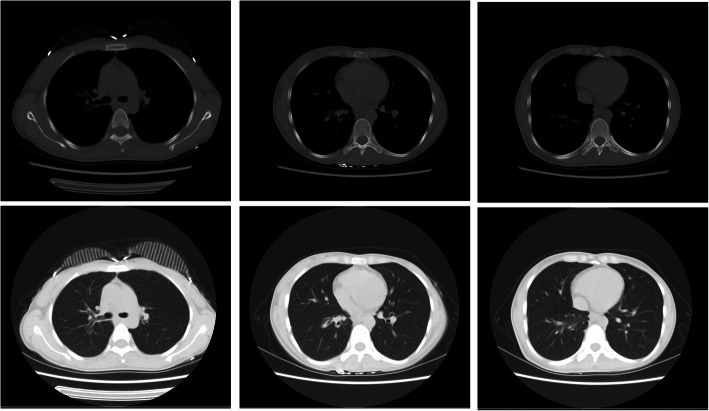
Chest CT of the patient. The right upper lobe had minute nodules; the right lower lobe had proximal segmental lung insufficiency; the right paraspinal pleura was slightly thickened; the mediastinal lymph nodes were increased; and the wall of the esophagus was limited and slightly thickened. No swollen lymph nodes on cervical region and bilateral supraclavicular fossa is evident

**Fig. 3:**
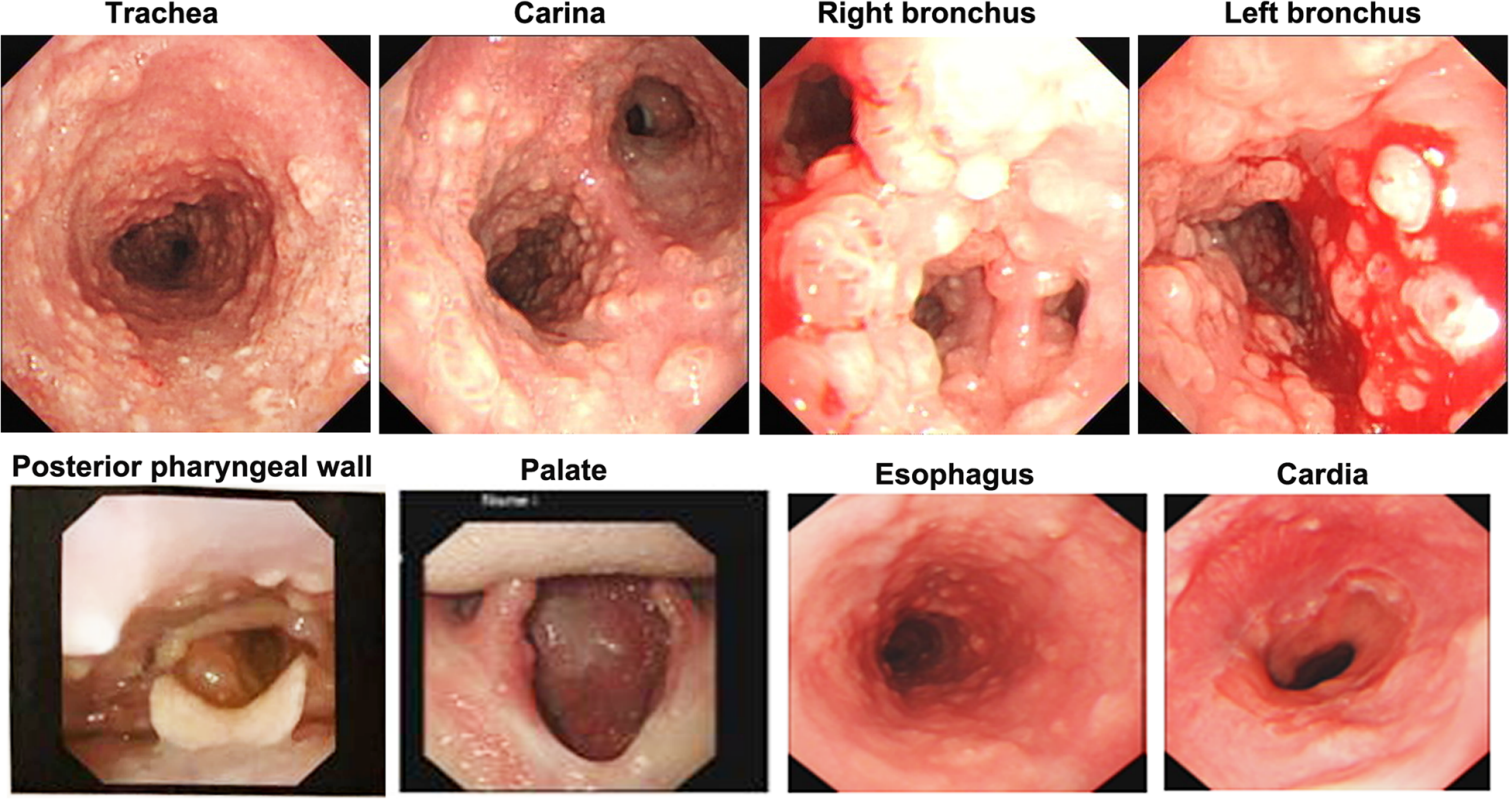
Fiberbronchoscopy showed that a large number of small bulges are densely distributed in the whole trachea, carina and bronchi covered withmanywhite viscous secretions. The surface of these bulges is smooth. Blood vessels are abundant and easy to bleeding. The openings of the right middle lobe, the right lower lobe and the left upper lobe bronchial were markedly narrowed. Laryngoscopy and gastroduodenoscopy showed that abundant yellow-white tuberculous uplifts were widely distributed in the posterior pharynx wall and inferior segmental esophagus

On examination, cell counts of white blood (12.12×10^9/L, normal range 3.5–9.5×10^9/L), neutrophils (8.27×10^9/L, normal range 1.8–6.3×10^9/L) and eosinophil (1.27×10^9/L, normal range 0.02–0.52×10^9/L) increased. Additionally, the ESR (34 mm/H, normal range 0-20 mm/H), hs-CRP (8.1 mg/L, normal range 0-1 mg/L) and IgE (178.8 IU/ml, normal range 0–100 IU/ml) also increased. Other laboratory workup including liver and renal functions, anti-cardiolipid antibody, antinuclear antibody, antistreptococcal O titers, rheumatoid factors, thyroid function, HIV antibody quantification did not reveal any abnormalities. No obvious abnormality was found in abdominal viscera and vascular ultrasound, cardiac function and electrocardiogram. There was no significant change in chest CT compared with the last CT (Fig. 2). To explore the etiology, we performed bronchoscopy to collect alveolar lavage fluid and biopsy. Fiberbronchoscopy showed that a large number of small bulges were densely distributed in the whole trachea, carina and bronchi, covered with more white viscous secretions. The surface of these bulges was smooth. Blood vessels were abundant and easy to bleed. The openings of the right middle lobe, the right lower lobe and the left upper lobe bronchial were markedly narrowed (Fig. 3). The smear and culture of bronchial lavage fluid didn’t show any evidence of bacteria, fungi, or tuberculosis. Next generation sequencing (NGS) technology only found a low copy number of haemophilus parainfluenzae (191) and rothia aeria (163). Laryngoscopy and gastroduodenoscopy showed that abundant yellow-white tuberculous uplifts were widely distributed in the posterior pharynx wall and inferior segmental esophagus (Fig. 3).

**Fig. 4:**
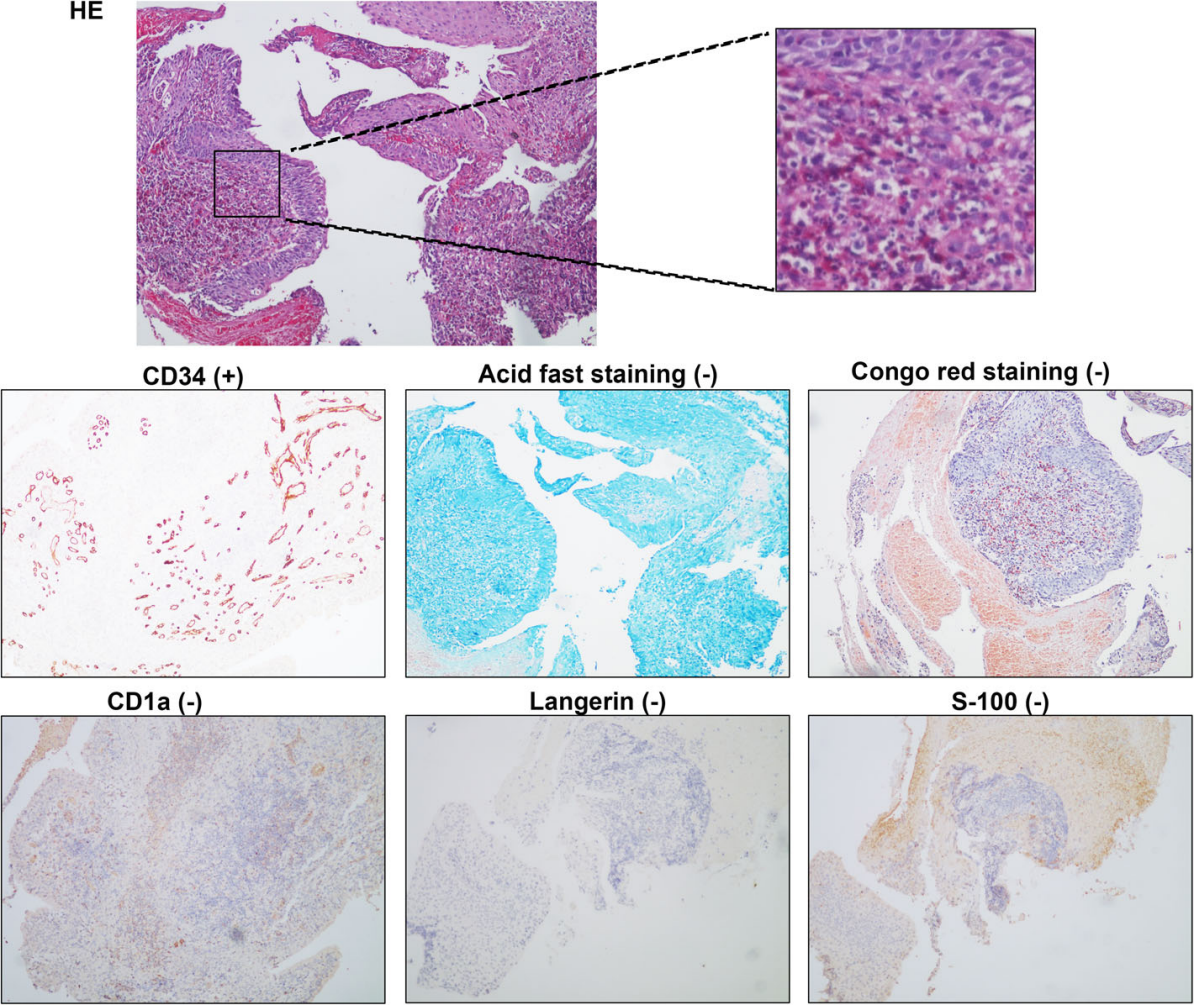
Biopsy specimens of bronchial nodules showed that part of the respiratory epithelium is squamous metaplasia with granulation tissue proliferating below, with numerous neutrophils and eosinophils infiltrating. No caseous necrosis, granulomatous lesions and tumor were observed in the sections. Immunohistochemical hinted that acid fast staining (−), congo red staining (−), CD34 (+), S-100 (−), CD1a (−), Langerin (−)

**Fig. 5:**
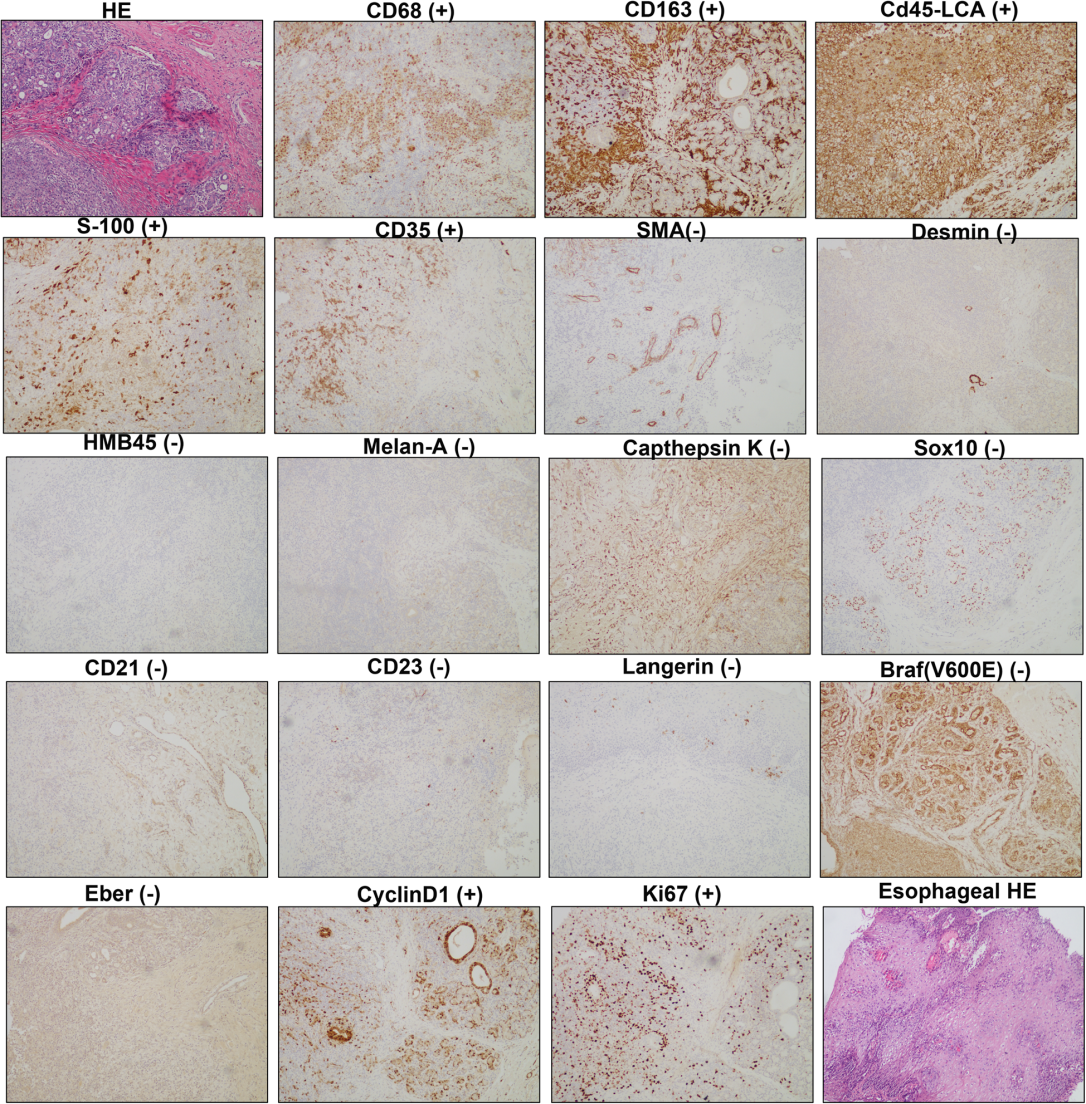
Biopsy specimens of oral nodules showed that a large amount of histiocytosis (some were foam cells) and neutrophil infiltration (the formation of a small abscess), with a small amount of eosinophils and lymphocytes infiltration in the subepithelial and salivary gland tissues without any caseous necrosis, obvious granulomatous lesions and neoplastic lesions. Immunohistochemical showed that CD163(+), CD68(+), CD45-LCA(+), S-100(+), CD35(+), SMA(−), desmin(−), HMB45(−), Melan-A(−), Cathepsin K(−), SOX10(−), CD21(−), CD23(−), Langerin(−), mutated Braf (V600E)(−), Eber(−), CyclinD1(+), Ki67(+). Biopsy specimens of esophageal nodules only found that numerous neutrophils and eosinophilic granulocyte infiltration

Biopsy specimens of bronchial nodules showed that part of the respiratory epithelium was squamous metaplasia with granulation tissue proliferating below, with numerous neutrophils and eosinophils infiltrating. No caseous necrosis, granulomatous lesions and tumor were observed in the sections. Immunohistochemical hinted that acid fast staining (−), congo red staining (−), CD34 (+), S-100 (−), CD1a (−), Langerin (−) (Fig. 4). Biopsy specimens of oral nodules showed a large amount of histiocytosis (some were foam cells) and neutrophil infiltration (the formation of a small abscess), with a small amount of eosinophils and lymphocytes infiltration in the subepithelial and salivary gland tissues without any caseous necrosis, obvious granulomatous lesions and neoplastic lesions (Fig. 5). Immunohistochemical showed that CD163(+), CD68(+), CD45-LCA(+), S-100(+), CD35(+), SMA(−), desmin(−), HMB45(−), Melan-A(−), Cathepsin K(−), SOX10(−), CD21(−), CD23(−), Langerin(−), mutated Braf (V600E)(−), Eber(−), CyclinD1(+), Ki67(+) (Fig. 5). Similarly, biopsy specimens of esophageal nodules only found numerous neutrophils and eosinophilic granulocyte infiltration (Fig. 5). Bone marrow examination showed no abnormality and rearrangement of FlP1L1/PDGFR gene which was associated with the negative result of primary hypereosinophilic syndrome (HES). These findings did not point specifically to a particular disease but were very similar to eosinophilia. She may be a specific phenotype of eosinophilia or may be a novel multisystem disease with respiratory system as the primary symptom, so we tried several treatment plans.

**Fig. 6:**
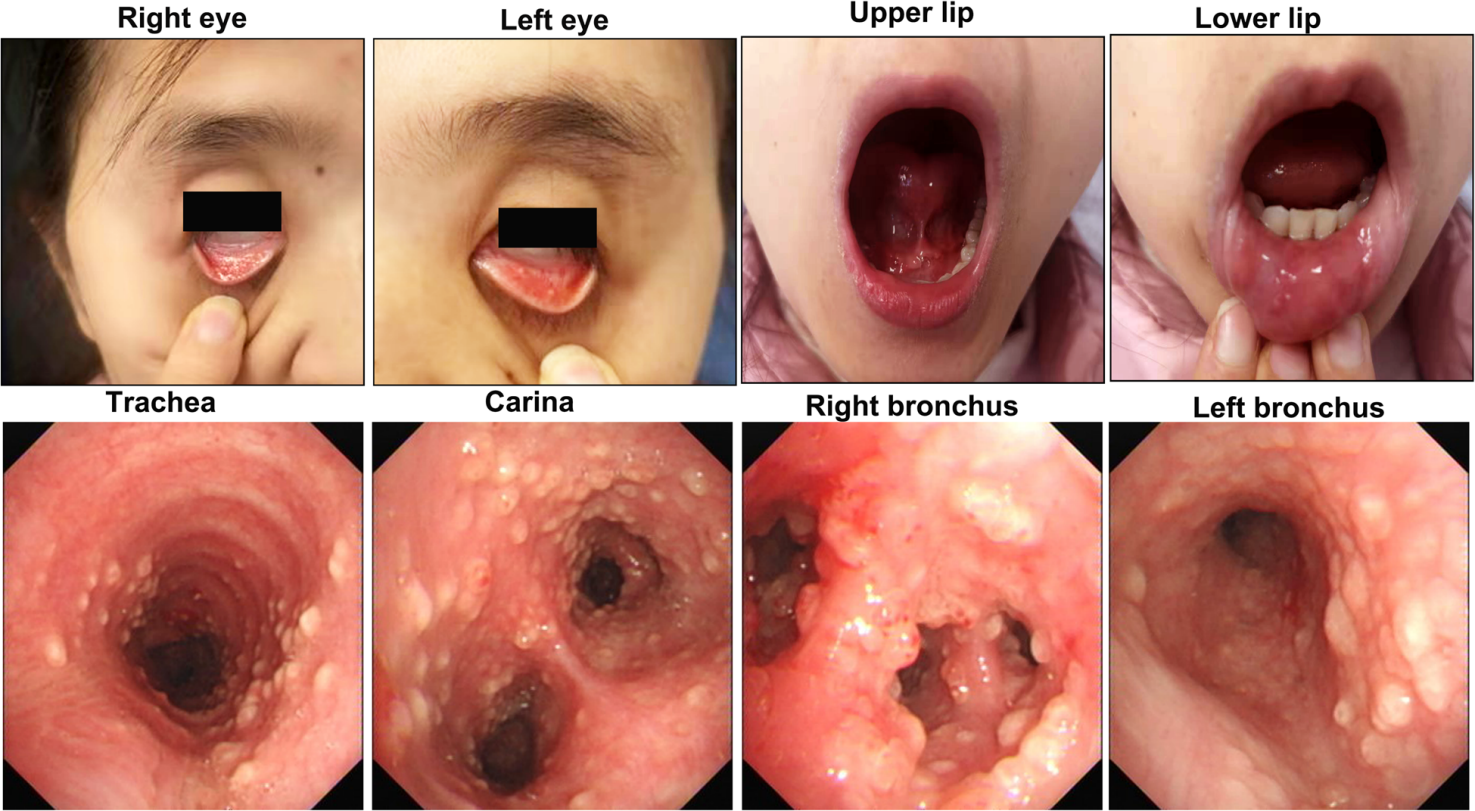
After a month of glucocorticoid treatment, oral and eye mucosa nodules has disappeared. The number of nodules on tracheal and bronchial mucosa also reduced

At first, the patient was treated with anti-infection (cefoperazone), anti-allergy (montelukast), anti-tussive (methoxyphenamine) and expectorant (ambroxol). However, these treatments showed few benefit after a month. In consideration of the extensiveness of the lesions, surgical resection was unfeasible. Thus, we tentatively gave her glucocorticoid (dexamethasone) 20 mg q.d, somac (pantoprazole) 40 mg q.d, caltrate D 300 mg q.d and arranged close follow-up. A month later, the symptoms of cough, expectoration, oral and eye mucosa nodules disappeared. The number of nodules on tracheal and bronchial mucosa also significantly reduced (Fig. 6).

In general, we found a strange disease in which patients presented recurrent cough and sputum, chest tightness, accompanied with extensive nodules of eye-mouth-bronchial mucosa. The biopsy specimen mainly showed inflammatory lesions (concluding a large number of neutrophils, moderate eosinophils and a small number of lymphocytes) and some granulation tissue proliferating, without caseoid necrosis and granuloma. Additionally, glucocorticoid rather than antibiotics was effective. Although it is not known whether this is related to the patient’s multisystem mucosal lesions, the patient also had a congenital dextrocardia and palatoschisis.

## Discussion and conclusions

Mucosal nodules are visible as rounded, elliptic, or irregular hyperplasia initiated by tumour, infection, autoimmunity, trauma or foreign matter [5]. Many noninfectious diseases, such as eosinophilia, amyloidosis, sarcoidosis, Wegener’s granuloma, langerhans cell histiocytosis, foreign bodies ect., are associated with the formation of mucosal nodules, especially significant bronchial lesions [4, 6–8]. Here we introduce the differential diagnoses and literature review of several rare bronchial diseases.

### Eosinophilia

The definition of eosinophilia is the absolute eosinophil count (AEC) in the peripheral blood is more than 0.5 × 10^9^/L with a percentage greater than 5% [9, 10]. The clinical features were varied, including weakness and fatigue, cough and fever, myalgias, dyspnea, and rash [11]. All organ systems are sensitive to sustained eosinophilia. Infiltrative dermopathy, pulmonary and gastrointestinal lesions accounted for the top three clinical manifestation in patients with hypereosinophilia [12]. Common causes of eosinophilia include helminthic parasite infections, varied types of adverse reactions to medications, and collagen vascular disease (Churg-Strauss syndrome, systemic lupus erythematosus), and primary myeloid tumor [13]. Additionally, we noticed a possible link between cleft lip/palate and eosinophilia. Several researches have indicated that eosinophilia was seen in 20–25% of cleft lip/palate cases [14, 15], but the evidences are not enough to verify their connection, so further studies are required to draw a clear conclusion. In the treatment of eosinophilia, the necessary therapy is removing causes. Systemic corticosteroids are the initial treatment at present when confronted with a patient with unexplained eosinophilia [16]. Although plasma eosinophilia and the presence of a large number of eosinophils in the lesion suggested that this case may be a secondary eosinophilia, extensive multisystem mucosal nodules in eosinophilia patients were not reported as prominent.

### Churg-strauss syndrome

In 1951, Churg and Strauss firstly described Churg-strauss syndrome (CSS) as a systemic antineutrophil cytoplasmic antibody (ANCA)-associated vasculitis. Hypereosinophilic disorder with lung involvement frequently occurs in people with asthma [17]. Based on the presence or absence of an ANCA, CSS were divided into two subtypes [18, 19]. Thus, ANCA positive is not requirement for CSS diagnosis. The pathological feature of CSS is a necrotizing vasculitis of medium to small sized blood vessels (veins and arteries) associated with eosinophilic infiltration around the vessels and adjacent tissues [17]. A combination of high-dose corticosteroids and cyclophosphamide is still the gold standard for the treatment of CSS [20]. Our patient had no history of asthma; the pathology reports of the patient didn’t suggest necrotizing small vasculitis; and ANCA test was negative, above which excluded the diagnosis of CSS.

### Tracheobronchial amyloidosis (TBA)

Tracheobronchial amyloidosis (TBA) is a rare disorder induced by abnormal deposition of excessive insoluble amyloid fibrils in the submucosa of respiratory tract [21]. Amyloid deposits can be focal, diffuse, or form localized masses, leading to various morphology. Based on the sites and severity of lesions, the clinical symptoms of TBA patients may vary from mild to severe, usually including flu-like symptoms, progressive dyspnea, and even respiratory failure [22]. Trachea, main bronchus, lobar bronchi, and segmental bronchi are the main lesion sites in TBA patients, while it has little impact on small airways and alveoli [23]. Chest X-ray is generally normal, but CT shows thickening of the tracheal walls, with narrowing of the tracheal lumen [24]. Bronchoscopy has a more important value in TBA diagnosis and can detect specific morphological changes, usually manifesting as single or multiple nodules or masses in tracheal and bronchial lumens, resulting in tracheal stenosis or occlusion [25]. Histopathological biopsy under bronchoscopy is listed as the gold standard for diagnosis of TBA. The characteristic change is that amyloid under tracheobronchial mucosa shows brick-red under light microscope and yellow-green birefringence under polarizing microscope after stained by Congo red [26]. Although the morphological changes under bronchoscopy of this patient seem to be similar with TBA, the pathological changes, especially Congo red negative, are not consistent with this disease.

### Pulmonary langerhans cell histiocytosis (PLCH)

Pulmonary langerhans cell histiocytosis (PLCH) is a rare granulomatous disorder characterized by the infiltration of dendritic cells sharing phenotypic similarities with langerhans cells in lesions [27]. Long-term smoking is an important driver. LCH may involve multiple organs at the same time, such as bones, skin, pituitary gland, lymph nodes, liver, spleen, etc. In the lungs, a nodular proliferation of langerhans cells often occurs in the bronchial mucosa and the alveolar septa, accompanied with the infiltration of eosinophilic granulocytes and destruction of the bronchial wall [28]. Langerhans cells can be selectively labeled by CD1a and langerin antibodies, which are the gold standard diagnostic modality [29, 30]. However, the immunohistochemistry of this patient revealed that both CD1a and langerin were negative which were not supported this disease.

### Wegener Granulomatosis (WG)

Wegener Granulomatosis (WG) is a rare autoimmune disease and is most common between 40 and 50 years of age. It is a systemic disease characterized by respiratory necrotizing, granulomatous vasculitis, glomerulonephritis, and vasculitis of other organs [31]. When skin and mucous membrane are involved, purpura, erythema, papules, pustules, subcutaneous nodules and ulcers are susceptible in lower limbs, but you can also find them on the trunk, upper limbs and maxillofacial region. Fibrolaryngoscopy or direct laryngoscopy showed typical subglottic annular stenosis with a surface of red brittle mucosa or erosive granulation tissue. Strawberry - like gingival hyperplasia is characteristic of WG in oral cavity [32]. CT findings were subglottic stenosis and thickening of the surrounding mucosa, as well as irregular changes in the trachea and bronchial walls and ulcerations [33]. The pathological features were the infiltration of neutrophils and mononuclear cells on the walls of pulmonary small blood vessels, with granulation of giant cells and multinucleated giant cells. Serology character of WG is the presence of anti-neutrophil cytoplasmic antibody (ANCA) to protease 3 (PR3, [34]). The results of ANCA test, renal function, urinalysis, imaging tests, and bronchoscopy of our patient didn’t support the diagnosis of WG.

### Sarcoidosis

Sarcoidosis is a multisystemic granulomatous disease of unknown etiology characterized by noncaseous necrotic granuloma. Between 30 and 60% of patients with sarcoidosis have no clinical symptoms and are diagnosed on X-ray during routine physical examination. Among those with symptoms, the most common ones are respiratory symptoms, rash, systemic symptoms, and musculoskeletal symptoms [35, 36]. The disease usually manifests as bilateral hilar lymph node enlargement, intrapulmonary infiltration, eye and skin infiltration, as well as liver, spleen and salivary glands [37]. Pulmonary imaging manifestations of sarcoidosis can be varied. Typical imaging features of chest involvement are mediastinal lymph node enlargement or bilateral hilar lymph node symmetry enlargement. Non-caseous necrotizing granuloma is the typical pathological feature of sarcoidosis [35], which was not consistent with our patient.

Treatments for respiratory system nodule diseases change over different diagnoses. For infectious nodules, adequate antibiotics, antifungal, or antiparasitics can help with recovery. Some inflammatory nodules could spontaneously alleviate without treatment [38, 39]. For eosinophilic angiocentric fibrosis and some fungal granulomas, surgical resection is a valid treatment. In the treatment of eosinophilia, sarcoidosis, Wegener’s granuloma, fungoid granuloma and other inflammatory nodule diseases, glucocorticoids are proved to be effective [38, 39].

In conclusion, we recognize the rarity of the disorder and lack of etiological diagnosis in our case. Generally, we can differentiate the type of nodule lesions on the basis of detailed medical history, comprehensive metabolic profile, imaging examination and biopsy specimen [39]. When the disorder type is difficult to determine, an attempt of glucocorticoid is worthy. In this rare case, the diagnosis of our patient remains unclear, she may be a specific phenotype of eosinophilia or may be a novel multisystem disease with respiratory system as the primary symptom. Clinical follow-up and gene detection are planned to provide accurate diagnosis and further treatments.

## Data Availability

The datasets generated for this study are available on request to the corresponding author.

## Abbreviations

PAP: Pulmonary alveolar proteinosis
LAM: Lymphangioleyomiomatosis
ESR: Erythrocyte sedimentation rate
Hs-CRP: High sensitivity C-reactive protein
HIV: Human immunodeficiency virus
NGS: Next generation sequencing
HES: Hypereosinophilic syndrome
AEC: Absolute eosinophil count
CSS: Churg-strauss syndrome
EGPA: Eosinophilic granulomatous polyvasculitis
ANCA: Antineutrophil cytoplasmic antibody
TBA: Tracheobronchial amyloidosis
PLCH: Pulmonary Langerhans cell histiocytosis
LCH: Langerhans cell histiocytosis
WG: Wegener Granulomatosis
